# Calcium, dairy, and prostate cancer

**DOI:** 10.1038/sj.bjc.6603680

**Published:** 2007-03-06

**Authors:** R P Heaney

**Affiliations:** 1 Creighton University, 2500 California Plaza, Omaha, NE 68178, USA

**Sir**,

In their paper on dairy, calcium, and prostate cancer risk, Koh *et al* (2006) in several places state incorrectly that an association with cancer risk is ‘biologically plausible for higher calcium intakes’. This is presumably based on the observation that high calcium intakes reduce circulating 1,25-dihydroxyvitamin D (1,25D), the active form of vitamin D, which, *in vitro*, inhibits the growth of prostate cancer cells and promotes their differentiation. All components of that assertion are correct, but it is incorrect to assume that lowered *serum* 1,25D would increase cancer risk. The critical compartment in which 1,25D is acting is intracellular, and the principal mechanism by which that concentration rises is by intracellular synthesis of 1,25D, not by transfer of 1,25D from the serum.

It is true that several authors (Schwartz *et al*, 1997; Giovannucci, 1998) have proposed that *serum* 1,25D is the active agent, but such proposals were made before there was full recognition of the autocrine role of 1,25D and of its synthesis from circulating 25(OH)D in the target tissues concerned. In a completely different model system (the innate immune response), Liu *et al* (2006) recently showed that the first response by a macrophage to an antigenic stimulus was the expression of the 1-*α*-hydroxylase, precisely to convert 25(OH)D to 1,25D, and that full expression of the immune response was dependent upon an adequate serum concentration of 25(OH)D.

As there is no known association between serum 25(OH)D and calcium intakes across the ranges commonly encountered, there is no longer any reason to consider a connection between calcium intake and prostate cancer risk as ‘biologically plausible’. In fact, understanding that it is the serum 25(OH)D concentration that is critical helps to explain why African Americans have high prostate cancer risk despite high circulating levels of 1,25D. By contrast, their serum 25(OH)D levels are typically only about half that of Caucasians. As serum 25(OH)D concentration is rate-limiting for the intracellular synthesis of 1,25D, it is most likely because of their low 25(OH)D levels that African Americans are at a disadvantage with respect to prostate cancer risk.

Thus, the study's null finding is hardly surprising.
